# Change in patterns of hospitalization for influenza during COVID‐19 surges

**DOI:** 10.1111/irv.12900

**Published:** 2021-08-23

**Authors:** Sindhaghatta Venkatram, Anuhya Alapati, Arundhati Dileep, Gilda Diaz‐Fuentes

**Affiliations:** ^1^ Division of Pulmonary and Critical Care Medicine BronxCare Health System, Affiliated with Icahn School of Medicine at Mount Sinai New York NY USA

**Keywords:** COVID‐19, influenza, mechanical ventilation, mortality, pneumonia

## Abstract

**Background:**

Hospitalization due to influenza has been stable in recent years. In March 2020, New York was an epicenter for coronavirus disease 2019 (COVID‐19). Because influenza and COVID‐19 present similarly, there were serious concerns that coinfection of these viruses would burden the healthcare system. We compared incidence and outcomes of patients hospitalized with influenza before and during COVID‐19 (seasons 2017–2021).

**Methods:**

We conducted a retrospective study evaluating hospitalized patients with influenza. Four influenza seasons were evaluated, 2017–2021, pre‐ and during COVID‐19 pandemic. We compared incidence of influenza and clinical outcomes across the seasons.

**Results:**

We found 412 patients hospitalized due to influenza in the study period; 394 had influenza, and 18 had both influenza and COVID‐19 infections. Demographics across the four influenza seasons were comparable; the cohort was predominantly female (61%) and had an average age of 60 years old. Comorbid conditions were common. No outcome differences were found for patients with influenza when comparing influenza seasons prior to and during the COVID‐19 pandemic. The mortality for the entire cohort was 6.5%. During the COVID‐19 pandemic, there were 18 (4.4%) influenza patients coinfected with COVID‐19 and 32 (7.8%) patients with bacterial super infection. Predictors of mortality in patients with influenza included presence of shock, heart failure, bacterial pneumonia, and use of mechanical ventilation. Coinfection with COVID‐19 did not increase mortality.

**Conclusion:**

We observed a significant decrease in the incidence of hospitalization due to influenza during the COVID‐19 pandemic. Clinical presentations and outcomes for patients with influenza remain stable. Being aware of possible increased mortality for patients with both influenza and bacterial pneumonia is important. Although coinfection with COVID‐19 did not increase mortality in influenza patients, identifying the specific virus responsible for infections has major therapeutic implications.

## 1 INTRODUCTION

Influenza viruses are members of the family Orthomyxoviridae. There are four genera of this family; however, only genera A and B are clinically relevant for humans and responsible for influenza. While seasonal influenza viruses are detected year‐round in the United States, influenza viral infections are most common during fall and winter. The exact timing and duration of influenza seasons vary, but usually, influenza activity begins to increase in October and peaks between December and February. Influenza activity can last as late as May.^1^

New York City (NYC) was an epicenter of the coronavirus disease 2019 (COVID‐19) outbreak in the United States during spring 2020.^2^ The clinical, epidemiological, and radiological features of COVID‐19 share many similarities with influenza. As COVID‐19 cases trended down in summer 2020, the medical community was seriously concerned that the upcoming influenza season (2020–2021) would overburden healthcare resources already strained by COVID‐19.

This study presents the influenza activity in our inner‐city hospital and compares influenza seasons before and throughout the COVID‐19 pandemic, seasons 2017–2021. We report outcomes for patients with influenza as well as for patients with dual infection of influenza and COVID‐19. Given that outcome data for patients with dual infections due to influenza and COVID‐19 are scarce, this study can serve as a valuable resource for the influenza community.

## 2 MATERIALS AND METHODS

### 2.1 Study design and patients

We conducted a retrospective cohort study at the 972‐bed community teaching hospital BronxCare Hospital System (BCHS) in South and Central Bronx. We included all adult patients admitted to the hospital for influenza infection who presented with respiratory symptoms and a positive nasal swab for influenza A or B rapid antigen test. The study period was October 1, 2017, to April 30, 2021.

Patients were divided into four groups based on influenza season as follows:

Period 1: October 1, 2017, to April 30, 2018.
Period 2: October 1, 2018, to April 30, 2019.
Period 3: October 1, 2019, to April 30, 2020.
Period 4: October 1, 2020, to April 30, 2021.

We compared outcomes across the four influenza seasons. For the last two seasons, which coincided with the COVID‐19 pandemic, we compared outcomes for patients with only influenza and for patients with influenza and COVID‐19 infections. COVID‐19 infections were identified using the Roche Cobas SARS‐CoV‐2 test.

The primary outcome was incidence of influenza in the four time periods. Secondary outcomes were needed for critical care admission, length of hospital stay, development of shock, use of mechanical ventilation, and mortality from all causes during hospital admission.

Ethics approval: Our study protocol was approved by the Institutional Review Board (approval number 004082105). We followed the amended Declaration of Helsinki.

### 2.2 Data abstraction

All data were retrospectively extracted from medical records, including demographic, clinical, and laboratory information. Radiology reports of chest radiographs (CXR) were used to collect radiological findings.

### 2.3 Statistical analysis

For the continuous variables, the one‐way analysis of variance (ANOVA) test was conducted to compare across the four time periods. Mean and standard deviation are reported in the tables for continuous variables. For the comparison of categorical variables across the four time periods, we conducted the chi‐squared test of independence. The overall *p* value refers to the chi‐squared test used to compare all four clinical types.

We completed a multivariate regression analysis with mortality as the predicted variable and the parameters as the explanatory variables. Because the dependent variable, mortality, was dichotomous, a binary logistic regression was conducted to establish the significant predictors. First, the omnibus test was carried out to determine whether the logistic null model was better than the final model with all the predictor variables. Lastly, to validate the model goodness‐of‐fit, the Hosmer and Lemeshow test was carried out. The results established that the model did have a good fit. The level of significance used in our study is *α* = 0.05.

## 3 RESULTS

A total of 412 patients with influenza were identified during the study period. Comparison of the number of cases in New York State, Bronx County, and BCHS as well as the number of influenza tests ordered at BCHS is shown in Table 1. There was a decline in the number of influenza cases in 2020–2021 compared with other time periods (*p* < 0.001). The number of influenza tests per 100 admissions declined between the 2019–2020 and 2020–2021 seasons, whereas the number of influenza tests performed during the 2017–2018, 2018–2019, and 2020–2021 seasons was comparable.

**TABLE 1:** Trend of influenza cases per season and comparison with state and county

	Influenza seasons			
	2017–2018	2018–2019	2019–2020	2020–2021
Influenza cases NY State	126,789	106,758	157,758	4460
Influenza cases in Bronx County	11,749	10,902	20,962	150
Influenza cases at BCHS	87	132	187	6
Number of influenza tests performed BCHS	3253	3641	5825	2862
Number inpatient admissions at BCHS	15,475	15,218	14,935	13,110
Influenza test/100 admissions at BCHS	21	23.9	39	21.8

Abbreviation: BCHS, BronxCare Health System.

Comparison of demographics, select comorbid conditions, and clinical presentation are shown in Table 2. In the influenza COVID‐19 overlap season (2020–2021), there were more African American and asthmatic patients. Additionally, the days of symptoms prior to hospitalization were longer, fewer patients presented with fever, and fewer patients had constitutional symptoms during the 2020–2021 influenza COVID‐19 overlap season.

**TABLE 2:** Comparison of demographic and clinical presentation by influenza season

Characteristics	Influenza seasons				*p* value^a^
	2017–2018	2018–2019	2019–2020	2020–2021	
	*n* = 87	*n* = 132	*n* = 187	*n* = 6	
Age (mean ± SD)	62.8 ± 15	57.1 ± 18	56.9 ± 184	63.8 ± 16	0.053
Female sex	49 (56.3)	83 (62.9)	116 (62.0)	3 (50.0)	0.710
Race/ethnicity					
White	5 (5.6)	3 (2.3)	0 (0.0)	0 (0.0)	0.005
African American	27 (31.0)	43 (32.6)	70 (37.4)	3 (50.0)	0.005
Hispanic	22 (25.3)	48 (36.4)	78 (41.7)	1 (16.7)	0.005
Other	33 (37.9)	38 (28.89)	39 (20.7)	2 (33.3)	0.005
Received influenza vaccine	22 (25.3)	29 (21.9)	36 (19.3)	1 (16.7)	0.705
Hypertension	62 (71.3)	86 (65.2)	121 (64.7)	3 (50.0)	0.585
Diabetes mellitus	35(40.23)	49 (37.1)	73 (39.0)	1 (16.7)	0.695
Heart failure	20 (22.9)	15 (11.4)	28 (14.9)	1 (16.7)	0.140
Chronic obstructive airway disease	24 (27.7)	27 (20.5)	30 (16.0)	0 (0.0)	0.088
Asthma	16 (18.4)	43 (32.6)	64 (34.2)	1 (16.7)	0.044
Obstructive sleep apnea	4 (4.6)	8 (6.1)	14 (7.5)	0 (0.0)	0.732
Malignancy active	8 (9.2)	5 (3.8)	16 (8.6)	0 (0.0)	0.277
Clinical presentation					
Days of symptoms	4.54 ± 3	3.3 ± 3	3.25 ± 2.6	3.67 ± 1.6	0.003
Fever	53 (60.9)	103 (78.0)	126 (67.4)	3 (50.0)	0.030
Constitutional symptoms	54 (62.1)	129 (97.7)	173 (92.5)	3 (50.0)	<0.001

^a^ For the continuous variables, to compare across the four time periods, the one‐way ANOVA test was conducted, and for the comparison of categorical variables across the categorical time periods, the chi‐squared test of independence was performed.

Table 3 shows pertinent laboratory and radiological investigations. We detected no significant differences between the influenza seasons for various biomarkers including D‐dimers, serum creatinine, platelets, and pro‐BNP. We did not observe significant differences in ejection fraction between the seasons. Finally, we did not observe significant differences in the percentage of patients presenting with normal or abnormal CXR between the influenza seasons. Dual infection with influenza and COVID‐19 was identified in 14 patients during the 2019–2020 season and four patients during the 2020–2021 season.

**TABLE 3:** Comparison of laboratory and chest imaging by influenza season

	Influenza seasons				*p* value^a^
	2017–2018	2018–2019	2019–2020	2020–2021	
	*n* = 87 (%)	*n* = 132 (%)	*n* = 187 (%)	*n* = 6 (%)	
D‐Dimers (ng/ml)	356.2 ± 233.4	3291.2 ± 9359.0	621.1 ± 660.2	1194.2 ± 1588.4	0.327
Serum creatinine (mg/dl)	1.45 ± 1.6	1.44 ± 1.4	1.44 ± 1.5	2.78 ± 1.7	0.185
Platelet (k/ul)	221.4 ± 80.8	198.9 ± 76.9	212.6 ± 80.4	155.3 ± 73.2	0.062
Pro‐BNP (pg/ml)	7690.7 ± 15469.8	5528.9 ± 18097.7	6479.4 ± 35038.5	866.2 ± 1126.5	0.918
Ejection fraction (%)	55.9 ± 19.5	60.9 ± 14.2	60.14 ± 14.6	51.3 ± 12.6	0.207
Influenza A	71 (81.6)	126 (95.5)	154 (82.4)	5 (83.3)	0.028
Influenza B	15 (17.2)	6 (4.6)	32 (17.1)	1 (16.7)	
Influenza A and influenza B	1 (1.2)	0 (0.0)	1 (0.5)	0 (0.0)	
COVID test positive			14 (7.5)	4 (66.7)	
Chest radiograph					
Unilateral infiltrates	14 (16.1)	22 (16.7)	27 (14.4)	1 (16.7)	0.081
Bilateral infiltrates	25 (28.7)	19 (14.4)	40 (21.4)	4 (66.7)	
Pleural effusions	1 (1.2)	4 (3.0)	5 (2.7)	0 (0.0)	
Normal	47 (54.0)	87 (65.9)	115 (61.5)	1 (16.7)	

^a^ For the continuous variables, to compare across the four time periods, the one‐way ANOVA test was conducted, and for the comparison of categorical variables across the categorical time periods, the chi‐squared test of independence was performed.

Patients in the influenza COVID‐19 overlap season (2020–2021) had an increased need for intensive care unit (ICU) admission, use of mechanical ventilation, and a longer length of hospital stay. There was no difference in mortality between the four periods.

A total of 394 patients (93.6%) had only influenza, and 18 patients (4.4%) had both influenza and COVID‐19 infections. There was no difference in demographics or comorbid conditions between these groups. We found 32 patients (7.8%) with influenza who had microbiologically positive bacterial pneumonia.

Comparisons of primary and secondary outcomes are shown in Table 4. The overall mortality for hospitalized patients with influenza was 6.5% (range = 4.6–16.7%) during the seasons studied. We observed an increased need for mechanical ventilation and longer length of hospital stay in the 2020–2021 season; these data need to be interpreted with caution as the total number of influenza cases was low and some of these patients were also infected with COVID‐19.

**TABLE 4:** Comparison of outcomes by influenza season

	Influenza seasons				*p* value^a^
	2017–2018	2018–2019	2019–2020	2020–2021	
	*n* = 87 (%)	*n* = 132 (%)	*n* = 187 (%)	*n* = 6 (%)	
Admission to critical care	35 (40.2)	41 (31.1)	57 (30.5)	3 (50.0)	0.308
Mechanical ventilation	22 (25.3)	14 (10.6)	25 (13.4)	1 (16.7)	0.022
Hospital length of stay	8.38 ± 9.7	7.04 ± 11.3	7.06 ± 7.5	19.33 ± 16.1	0.013
Mortality	6 (6.9)	6 (4.6)	14 (7.5)	1 (16.7)	0.541

^a^ For the continuous variables, to compare across the four time periods, the one‐way ANOVA test was conducted, and for the comparison of categorical variables across the categorical time periods, the chi‐squared test of independence was performed.

Predictors of mortality in patients with influenza are shown in Table 5. Admission to ICU, shock, need for mechanical ventilation, length of hospital stay, low ejection fraction, presence of secondary bacterial pneumonia, and unilateral infiltrates on chest radiograph were predictive of mortality. Normal radiograph was associated with lower mortality.

**TABLE 5:** Multivariate model: Predictors of mortality in patients with influenza

	OR	95% CI	p
Shock	4.7	1.62–13.96	<0.001
Mechanical ventilation	2.0	1.20–3.50	<0.001
Bacterial pneumonia	3.2	1.09–9.76	<0.001
Ejection fraction	2.3	1.02–5.32	<0.001
Unilateral infiltrates in chest radiograph	1.8	1.07–3.14	<0.001
Normal chest radiograph	0.4	0.22–0.95	<0.001
Intensive care unit admission	1.91	1.12–3.24	<0.001

## 4 DISCUSSION

Our study is consistent with data reporting a decrease in influenza infections during the COVID‐19 pandemic and provides information on influenza‐related hospitalization in a vulnerable, diverse ethnic population with multiple comorbidities in one of the poorest areas of NYC. Dual infection with COVID‐19 was rare and not associated with increased mortality in our cohort.

The reason for a decline in influenza cases could be the nonpharmacological interventions implemented to decrease exposure and transmission of COVID‐19. Influenza and COVID‐19 are both primarily spread via small, virus‐laced particles and respiratory droplets released when an infected person coughs, sneezes, talks, or simply exhales. A person can get infected either by inhalation or through physical contact, like handshaking or hugging, followed by touching their own nose or mouth. Many nonpharmacological epidemiological measures implemented during the COVID‐19 pandemic, including quarantining, wearing masks, social distancing, and frequent hand washing, are known to decrease influenza infections.^3^, ^4^, ^5^, ^6^ Furthermore, a recent study found that decreases in influenza infections were associated with the implementation and timing of COVID‐19‐related nonpharmacological interventions in China and the United States.^7^ These nonpharmacological interventions could account, at least in part, for the decrease in influenza infections in the 2019–2020 and 2020–2021 seasons. Finally, it is possible that some patients with influenza stayed home due to reluctance to seek emergency room care during the pandemic.

Although it would be intuitive to associate the lower number of influenza cases with a smaller number of tests performed during the COVID‐19 pandemic, our results do not support this notion. Lagacé‐Wiens et al. reported a decreased incidence of influenza during the COVID‐19 pandemic and reported that the weekly positivity rates declined from 20.16 to 0.11 during the COVID‐19 pandemic.^6^ In our institution, an influenza test is ordered for influenza‐like illness seasonally. Consistent with the state and county trends, influenza testing and cases were higher in 2019–2020 seasons. However, when we compared the 2020–2021 trend with 2017–2018 and 2018–2019 seasons, there were no statistical decrease in number of total tests ordered or tests ordered per 100 admissions. Therefore, it is unlikely that decreased testing accounts for the lower number of influenza cases in 2020–2021.

Age; comorbid illnesses such as atrial fibrillation, acute heart failure, pneumonia, malnutrition, and admission to intensive care; shock; and respiratory failure are predictors of mortality for patients with influenza.^8^, ^9^, ^10^, ^11^ Additionally, certain laboratory findings, including a change in serum D‐dimer values, can serve as a predictor of mortality.^12^ An association between low platelets and mortality has been reported in avian flu.^13^ Our study reveals no association between comorbid conditions and mortality; however, our data support prior studies showing an increased influenza mortality in patients requiring ICU care, presence of shock, and need for mechanical ventilation.^8^ The presence of COVID‐19 pandemic and overlap of COVID‐19 in the two last seasons did not change the association of mortality with ICU admission, shock, and need for mechanical ventilation.

Comparison of clinical outcomes in critically ill patients hospitalized with COVID‐19 or influenza has been published.^14^ Stowe et al. reported that co‐circulation of influenza and COVID‐19 viruses could have a significant impact on morbidity, mortality, and health service demand.^15^ Our study contributes new and unique data to the field because we evaluated the last four seasons of influenza in our inner‐city hospital including an overlap of two COVID‐19 waves. Furthermore, we evaluated COVID‐19 as a secondary infection and confounders of mortality. The presence of COVID‐19 coinfection does not seem to impact mortality in our patient population; we hypothesize that this could be due to the few cases of coinfections in our cohort.

Influenza coinfections have been reported with bacterial infections; rates range from 2% to 65% with Streptococcus pneumonia been the most common bacterial pathogen.^16^, ^17^ Coinfecting bacterial pathogens are a major cause of morbidity and mortality in patients infected with influenza. Furthermore, coinfections are associated with both pandemic and seasonal influenza virus illness.^18^, ^19^, ^20^ Tissue examination of bacterial coinfections in lung tissue specimens from fatal cases of the 2009 pandemic influenza A (H1N1) in United States between May and August 2009 revealed Streptococcus pneumonia and *Staphylococcus aureus* as the two most common coinfected organisms.^21^ Common causes of bacterial infections associated with influenza are Streptococcus pneumonia, *S. aureus*, *Pseudomonas aeruginosa*, *Streptococcus pyogenes*, *Haemophilus influenzae*, *Klebsiella pneumoniae*, and Mycoplasma pneumonia.^16^

Influenza infection is associated with increased hospital morbidity and mortality in patients with heart failure.^22^, ^23^ Our study revealed a clear association between ejection fraction and mortality in patients with heart failure.

The presence of chest radiological abnormality is predictive of worse outcomes in patients with influenza; pulmonary infiltrate, consolidation, and effusion are associated with the worst outcomes.^24^ Aviram et al. reported that initial chest radiography may have significance in helping predict clinical outcome, but normal initial radiographs cannot exclude adverse outcome.^25^ In their study, extensive involvement of both lungs, evidenced by the presence of multizonal and bilateral peripheral opacities, was associated with adverse prognosis. In our study, a normal CXR on admission was associated with decreased mortality, and unilateral infiltrates were associated with increased mortality. Bilateral infiltrates or pleural effusions had no predictive value.

Our study shows the trends of influenza admissions in an inner‐city hospital in NYC over four seasons: two before COVID‐19, one with partial overlap of influenza and COVID‐19, and the last one during both influenza and COVID‐19. This is a real‐life study on the behavior of influenza seasons coinciding with the two COVID‐19 waves. Like other studies, we found a significant decrease in influenza infections after the emergence of COVID‐19 infections. The presence of coexisting infections with COVID‐19 was not predictive of mortality. It is expected that the next influenza season may have an increased number of hospitalizations as the numbers of COVID‐19 infections decrease and non‐pharmacological measures are relaxed or eliminated.

Identifying the viral etiology for respiratory infections and pneumonia has major clinical and therapeutic implications. Although systemic steroids decrease mortality in patients with COVID‐19 who require supplemental oxygen or mechanical ventilation due to hypoxia,^26^ current clinical practice guidelines from the Infectious Diseases Society of America advise against the use of corticosteroids in influenza.^27^ Therefore, it is advisable to test patients with influenza‐like illness early for both influenza and COVID‐19, especially if COVID‐19 steroid treatment is being considered.

The strengths of the study include real‐life comparison of four consecutive influenza seasons, including two waves of COVID‐19 pandemic. Influenza rates paralleled the rates reported in New York State and the Bronx Borough. The study was performed in one of the poorest areas of NYC, which included a diverse population of minority groups at a social disadvantage who were at the highest risk for morbidity and mortality during the pandemic.^28^ This demographic also has lower rates of influenza vaccination.^29^ To the best of our knowledge, limited research has been done examining the outcomes of coinfections between influenza and COVID‐19. Limitations of the present study include the retrospective nature of the study as well as that it was performed in a single center. Thus, further research is necessary to support widespread changes in practice.

## 5 CONCLUSION

Influenza infections have shown a downward trend after the emergence of COVID‐19. Predictors of poor outcomes and mortality in hospitalized patients with influenza remain basically unchanged throughout seasons in our inner‐city community. It seems that the risk of coinfection with another virus like COVID‐19 is small and does not increase mortality.

We anticipate that COVID‐19 will remain a significant pathogen in the upcoming influenza seasons. Early identification of viral etiology has major therapeutic implications; antiviral treatments are available for influenza patients, and selected patients with COVID‐19 could benefit from steroids.

Patients with dual infection will present a management challenge for the clinicians. However, with the limited available data on coinfections, it is too early to suggest specific treatments. Prevention of infection will continue playing a significant role in our high‐risk population. As we approach the coldest months of the year, vaccinating for COVID‐19 and influenza as well as continuing with some nonpharmacological measures is of the utmost importance.

## CONFLICT OF INTEREST

There are no conflicts of interest declared from all the authors. The authors have no disclosure.

## AUTHOR CONTRIBUTIONS

**Sindhaghatta Venkatram:** Conceptualization; formal analysis; investigation; methodology; supervision. **Anuhya Alapati:** Data curation; investigation. **Arundhati Dileep:** Data curation; investigation. **Gilda Diaz‐Fuentes:** Conceptualization; formal analysis; investigation; methodology; supervision.

### PEER REVIEW

The peer review history for this article is available at https://publons.com/publon/10.1111/irv.12900.

## Data Availability

The data that support the findings of this study are available from the corresponding author upon reasonable request and pending additional ethical approval.
